# Perceived barriers to management of patients with diabetes mellitus and hypertension in primary care centers in Indonesia

**DOI:** 10.3389/frhs.2025.1715125

**Published:** 2026-01-15

**Authors:** Ni Made Hustrini, Endang Susalit, Merel van Diepen, Joris Ivo Rotmans

**Affiliations:** 1 Division of Nephrology and Hypertension—Department of Internal Medicine, Faculty of Medicine—Universitas Indonesia, Dr. Cipto Mangunkusumo National General Hospital, Jakarta, Indonesia; 2 Department of Internal Medicine, Leiden University Medical Center, Leiden, Netherlands; 3 Department of Clinical Epidemiology, Leiden University Medical Center, Leiden, Netherlands

**Keywords:** diabetes, health care providers, hypertension, perceived barriers, primary health care

## Abstract

**Objectives:**

This study aimed to explore provider knowledge, barriers, and opportunities for improving chronic disease management in Indonesian primary health care.

**Methods:**

A descriptive cross-sectional mixed methods study was conducted from December 1, 2022 to March 31, 2023 across 14 primary care centers in Jakarta. Multidisciplinary healthcare providers responsible in hypertension and diabetes mellitus (DM) care were involved. Data were collected using a semi-structured, interviewer-administered questionnaire consisting of both closed- and open-ended questions, structured across five domains: referral system knowledge, referral pathway implementation, hypertension management knowledge, DM management knowledge, and delivery of chronic disease care. Quantitative data were complemented with qualitative responses to provide contextual insight.

**Results:**

A total of 59 healthcare providers participated in the study, the majority of whom were female (89.8%). Participants included physicians (42.4%), nurses (35.6%), and midwives (10.2%). Most participants (71.4%) had over three years of practice. Notable knowledge gaps were identified in referral practices, diagnostic criteria, and monitoring protocols. Referrals were often delayed until complications occurred. Implementation of the national chronic disease (PROLANIS) program was suboptimal, particularly in patient selection and defining outcome targets. Screening for kidney complications was limited and medication availability was restricted. Poor adherence to national guidelines, short consultation times, and staffing shortages further hindered care and education efforts. Three major barriers—patient-related, provider-related, and structural—were identified.

**Conclusions:**

Improving chronic disease care in primary settings requires addressing provider knowledge gaps, strengthening referral systems, and enhancing PROLANIS implementation through targeted training and resource allocation.

## Introduction

Indonesia's ongoing epidemiological transition present major challenges to its healthcare system. While life expectancy increases up to 68.3 years in 2023 and productive-age population grows to 80%, the burden of non-communicable diseases (NCDs) continues to rise (1). As progress of interventions against communicable diseases remain slow, NCDs account for more than half of all deaths nationally, with stroke, ischemic heart disease, diabetes mellitus (DM), hypertension, and kidney diseases among the top ten causes (1). In 2019, 25% of 30-year-olds were likely to die from NCDs before the age of 70 (1). By 2021, nearly 250.000 deaths were attributed to DM, and 69% of cardiovascular death were linked to hypertension (2–4).

Within this broader NCD landscape, diabetes and hypertension are evident due to their high prevalence, substantial morbidity, and closely linked to complications such as cardiovascular disease and chronic kidney disease. Approximately 11% of Indonesia's population—roughly one in nine adults—has diabetes, with an estimated 74% of cases remaining undiagnosed and around 236,000 deaths linked to the disease (5). Hypertension affects about 40% of the population, a rate higher than the global average, and high systolic blood pressure is associated with about 69% of cardiovascular-related deaths (6).

To address these challenges, various national guidelines for diabetes and hypertension management have been introduced, supported by initiatives from the Ministry of Health and the national health insurance agency (BPJS Kesehatan). In 2014, BPJS launched PROLANIS—a community-based program targeting diabetes and hypertension at the primary care level. However, disease control remains poor (7); the 2023 National Health Survey reported that fewer than 20% of patients with DM and/or hypertension are well-controlled (8).

Primary care plays a central role in the management of diabetes and hypertension in Indonesia. With more than 10,000 primary care centers nationwide, these facilities form the backbone of chronic disease prevention, early detection, and long-term management (8). Despite past initiatives, outcomes remain suboptimal indicating that challenges extend beyond patient-related factors (7, 8). Emerging evidence highlights the importance of provider knowledge, clinical decision-making, and organizational dynamics in shaping the quality of chronic disease care (9–11).

Given these persistent gaps, a clearer understanding of primary care providers' knowledge, perceptions, and experiences in managing diabetes and hypertension is needed. Such insights are essential for strengthening existing programs, informing policy alignment, and improving the delivery of chronic care services. This study aims to assess primary care providers' knowledge and perceptions of diabetes and hypertension management in Indonesia and to explore barriers and opportunities to enhance care delivery at the frontline of the health system.

## Materials and methods

### Study design and setting

This descriptive cross-sectional mixed methods study was conducted from December 1, 2022 to March 31, 2023, across 14 primary care centers in Jakarta, Indonesia. While primarily quantitative, it included a qualitative component contextualized healthcare provider's experiences and challenges.

### Participants

#### Inclusion criteria

Respondents were multidisciplinary healthcare provider–including physicians, nurses, midwives, pharmacist, dentist, nutritionist, or other relevant professionals–responsible for hypertension and DM management. Prior to data collection, we coordinated with site coordinators to identify healthcare providers involved in chronic disease management, particularly DM and hypertension. Written informed consent was obtained from all participants.

### Study outcomes

We aimed to assess knowledge of healthcare providers of referral systems, diabetes and hypertension management, and perceived barriers to care.

### Data sources/measurements

Data were collected using a semi-structured, interviewer-administered questionnaire consisting of both closed- and open-ended questions, structured across five domains:

Referral system knowledge
Referral pathway implementation
Hypertension management knowledge
DM management knowledge
Delivery of chronic disease care

### Quantitative component

Closed-ended questions assessed knowledge and practice patterns using multiple-choice and dichotomous formats. Each correct answer received 1 point, and total scores were categorized into three levels:

Referral system knowledge (max score = 10): low (1–3), moderate (4–7), high (8–10)
Hypertension knowledge (max score = 21): low (1–7), moderate (8–14), high (15–21)
Diabetes knowledge (max score = 16): low (1–5), moderate (6–10), high (11–16)

The questionnaire also assessed antihypertensive and antidiabetic medications availability, adherence to national guidelines, health education provision, treatment target achievement, average consultation time, and reported barriers to care (Supplementary Tables S1–S4).

### Qualitative component

An open-ended question explored perceived barriers in managing hypertension, DM, and referral process—particularly regarding PROLANIS. Responses were transcribed and categorized into three main themes: patient, provider, and organizational/structural factors.

### Demographic variables

Demographic data included gender, center location, professional role, education, and years of clinical experience.

Questionnaire was developed based on PROLANIS guideline and national consensus on hypertension and DM management (12–14). The questionnaire reviewed by two internist-nephrologists and two public health experts, and was pilot-tested on ten healthcare workers for clarity and relevance.

### Sample size and recruitment

The exploratory purpose of study was designed to provide an initial understanding of a complex topic, identify key variables, and establish a foundation for future, more specific research questions. No formal sample size calculation was performed. Instead, a pragmatic approach was taken by including all eligible healthcare providers at 14 participating centers.

### Data analysis

Quantitative data were analyzed using SPSS version 29 and presented as frequencies, percentages, and bar charts, using complete case analysis. For the qualitative component, open-ended responses were reviewed, coded, and organized into three thematic categories. We employed a basic thematic analysis rather than discourse analysis or other formal qualitative methodologies. The coding process involved identifying recurring ideas, grouping them into three overarching themes, and resolving any discrepancies through discussion among the research team. Although the qualitative component was descriptive and exploratory in nature, we followed standard steps of familiarization, coding, and theme development to ensure that the analysis was systematic and transparent.

This study followed STROBE guidelines for observational studies (15).

## Results

We included 59 healthcare providers, mostly female (89.8%), physician (42.4%), nurse (35.6%), or midwife (10.2%). Most subjects (71.4%) had more than 3 years of practice (Table 1).

**Table 1:** Baseline characteristics of study participants.

Parameters	*N* = 59
Gender, *n* (%)	
Female	53 (89.8)
Male	6 (10.2)
Profession, *n* (%)	
Doctor	25 (42.4)
Nurse	21 (35.6)
Midwife	6 (10.2)
Dentist	3 (5.1)
Pharmacist	2 (3.4)
Nutritionist	2 (3.4)
Years of practice, *n* (%)	
≥3 years	40 (71.4)
<3 years	16 (28.6)
*Missing data, n (%)*	3 (0.05)
Level of education, *n* (%)	
Bachelor degree	44 (74.6)
Post-graduate	15 (25.4)

Most subjects (65%) demonstrated moderate referral knowledge (RK), with only 34% high. Major gaps appeared across six key areas: 84% misunderstood DM referral criteria (RK2) with 62% preferred referral should occur only after chronic complications develop, rather than after failing to meet glycemic targets within three months of oral therapy. In RK5, 78% misidentified vertical referral criteria, and 67% assumed referral was unnecessary if resources were adequate, disregarding needs for specialized interventions. Referral priorities for chronic diseases (RK6) were misunderstood by 45%, and 25% chose referral for sudden blood pressure spikes without specialist indication (RK7). PROLANIS knowledge assessment (RK8–10), 25% misidentified the target population, confusing type 2 DM and hypertension with type 1 DM or obesity, and 36% overestimated the Controlled PROLANIS Participant Ratio (CPPR) target (Supplementary Figure S1).

In hypertension knowledge (HK), 78% showed high and 22% moderate levels. Significant gaps appeared across six areas: 26% were unaware hypertension is often asymptomatic (HK2), 49% incorrectly believed systolic hypertension alone poses greater cardiovascular risk than diastolic (HK4), 51% disagreed measuring blood pressure twice per visit at five-minute intervals (HK5), and 35% disagreed repeating measurements when discrepancies exceed 5 mmHg (HK6). Incorrect blood pressure targets for those over 65 (HK16) found in 42%. Kidney complications failed to recognize in 28% (HK17); 40% misidentified referral criteria for kidney dysfunction (HK19), selecting inappropriate indicators like hypoalbuminemia or eGFR 60–89 mL/min/1.73 m² instead of the correct threshold of eGFR <30. While 60% correctly identified nephrotoxic agents (HK21), some incorrectly included ACE-inhibitors and sodium bicarbonate (Supplementary Figure S2).

Most participants demonstrated moderate to high DM knowledge (DK, 36%–54%), yet significant gaps persisted across nine areas. About 69% were unaware that DM includes more than two types (DK1), and 57% disagreed with the correct timing for blood glucose monitoring (DK2). Nearly half (47%) misunderstood monitoring interval for type 1 DM (DK4), and 21% failed to identify common insulin side effects (DK6). Many (59%) incorrectly believed glucosuria alone confirms DM (DK10), while 64% showed poor understanding of risk factors (DK11) and early signs of diabetic foot complications (DK14). Diagnostic criteria (64%, DK15) and glucose targets (49%, DK16) for gestational diabetes were also poorly understood. Biguanides (60%) and sulfonylureas (28%) were the most available antidiabetics, with calcium-channel blockers, RAAS-inhibitors, and beta-blockers common among antihypertensives (Supplementary Figures S3–S5).

Table 2 shows that 94.4% of antidiabetic and antihypertensive drugs were covered by national insurance. Half of providers adhered to national guidelines, yet 58.9% believed fewer than half their patients met control targets. Patient education was inconsistent, mainly due to short consultations (under 10 min in 80.4% cases). Staffing shortages (42.9%), salary dissatisfaction (27.3%), and secondary jobs (23.2%) were reported. Early kidney screening was suboptimal.

**Table 2:** Assessment on health services provided by the primary care centers.

Health services	Category	*N* (%)
Are all of medicines covered by BPJS (Badan Penyelenggara Jaminan Sosial) Kesehatan or the national health insurance system?	Yes	51 (94.4)
	No	3 (5.6)
In treating patients, how often do you follow the national guidelines for the management of patients with diabetes mellitus or hypertension?	Always	27 (48.2)
	Frequently	20 (35.7)
	Rarely	4 (7.1)
	Never	5 (8.9)
How often do you educate patients about the side effects of medications and how to minimize them?	Always	33 (58.9)
	Frequently	14 (25)
	Rarely	7 (12.5)
	Sometimes/never	2 (3.6)
Do you frequently ask your patients about medication adherence?	Always/every meeting	43 (76.8)
	Frequently/few times	9 (16.1)
	Rarely/occasionally	1 (1.8)
	Sometimes/never	3 (5.4)
Besides pharmacological treatment, have you ever educated patients on diabetes mellitus or hypertension diet management?	Yes	52 (92.9)
	No	4 (7.1)
How often do you educate patients on how to check their blood sugar at home?	Always/every meeting	22 (39.3)
	Frequently/few times	24 (42.9)
	Rarely/occasionally	7 (12.5)
	Sometimes/never	3 (5.4)
How often do you educate patients to do blood pressure measurement at home?	Always/every meeting	24 (42.9)
	Frequently/few times	20 (35.7)
	Rarely/occasionally	10 (17.9)
	Sometimes/never	2 (3.6)
What is your blood sugar targets for diabetic patients?	Pre-prandial glucose 80–130 mg/dL	37 (66.1)
	Pre-prandial glucose <200 mg/dL	19 (33.9)
What is your blood sugar targets for diabetic patients?	BP <150/90 mmHg	6 (10.7)
	BP <140/90 mmHg	50 (89.3)
What percentage of diabetes mellitus and hypertension patients in your center are controlled/achieved treatment targets?	81%–100%	2 (3.6)
	51%–80%	21 (37.5)
	31%–50%	28 (50)
	0%–30%	5 (8.9)
Do you have any other job besides working at the primary care center?	Yes	13 (23.2)
	No	43 (76.8)
What is your average consultation time per patient?	More than 10 min per patient	11 (19.6)
	Less than 10 min per patient	45 (80.4)
In your opinion, is the incentive/salary given appropriate to your current workload?	Yes	40 (72.7)
	No	15 (27.3)
What barriers do your center most commonly encounter in managing patients?	Limited human resources	24 (42.9)
	BPJS system	10 (17.9)
	Facilities and infrastructure that are less supportive	6 (10.7)
	Limited service hours	12 (21.4)
	Others	4 (7.1)
How often do you perform urinalysis and kidney function tests (ureum, creatinine) for patients with diabetes mellitus and hypertension?	Very often	6 (10.7)
	Often	28 (50)
	Rarely	16 (28.6)
	Never	6 (10.7)

Challenges from an open-ended question were categorized into three main domains (Table 3).

**Table 3:** The perceived barriers of healthcare providers in the management of diabetes and hypertension.

Domain	Barriers
Patient-related factors	Low health awareness in the community, resulting in a low participation rate in the screening program.
	Lack of time.
	Fear of hospitals environment.
	Uncooperative patients: missed appointments, poor adherence to medications and lifestyle changes, refusal of referral.
	Lack of family support, leading to delays or refusal for referral.
	Long travel distance and associated transportation costs.
Health care providers-related factors	Lack of familiarity with guidelines.
	Lack of familiarity with referral system.
	Lack of familiarity with the national chronic disease management initiative (PROLANIS).
	Limited recognition or knowledge of kidney complication and associated management.
Organisational/structural factors	Inefficient administrative service.
	Extended waiting time.
	Absence of a well- organized referral systems.
	Lack of comprehensive clinical information system for referral documents.
	Insufficient staff to coordinate with relevant referral parties.
	Delayed referral feedback.
	Lack of follow-up or information regarding services received by patients.
	Patients must be referred back to the hospital to obtain the medication since the primary care physicians are not authorized to adjust prescriptions based on the treatment plan from the referral hospital.
	Insufficient training for health care providers in management of hypertension and diabetes.
	Laboratory tests must be conducted according to a set schedule.
	Some laboratory tests are not covered by the national health insurance system (BPJS Kesehatan).
	Prescription on certain medications requires supporting data, such as lab results, to be covered by the BPJS Kesehatan.
	Limited consultation time for comprehensive patient care.
	Shortage of healthcare workers.
	Out of supply for certain medications.
	Less reliable and less organized laboratory partners.
	The implementation of PROLANIS depends on the resources available in each region.
	Keynote persons and educational materials delivered were homogenous in PROLANIS program.
	The persons in charge were also responsible for other duty (program).
	Unable to remove patients who chose not to continue participating in PROLANIS from the system.

The qualitative findings help explain several patterns observed in the quantitative results. Patient-related barriers—such as low health awareness, poor adherence, missed appointments, fear of hospital environments, and long travel distances—correspond with the low rates of target achievement, where half of the respondents reported that only 31%–50% of their patients were controlled. This persists despite providers frequently asking about adherence (76.8%) and routinely offering education on medications and lifestyle.

Provider-related challenges, including limited familiarity with guidelines, referral pathways, PROLANIS procedures, and kidney complication management, help clarify why adherence to national guidelines was only moderate (48.2% always, 35.7% frequently). Short consultation times reported by 80.4% of providers further limit opportunities for patient education and comprehensive management.

Organizational and structural barriers—such as inefficient administrative processes, inadequate staffing, inconsistent referral feedback, laboratory scheduling constraints, and medication coverage policies under BPJS—align with the quantitative findings showing gaps in routine monitoring (28.6% rarely and 10.7% never conducting kidney function tests) and limited-service capacity. These system-level constraints also reflect the reported shortage of healthcare workers (42.9%).

Overall, the qualitative themes reinforce the quantitative data by illustrating how patient, provider, and system-level challenges collectively contribute to suboptimal chronic disease control in primary care settings.

## Discussion

Our study revealed significant knowledge gaps among healthcare providers in referral, hypertension, and diabetes management. Prevalent misunderstandings including timely referral, diabetes diagnostic criteria, and standard monitoring in blood pressure and glucose. Gaps in PROLANIS implementation involving incorrect patient selection and outcome targets. Kidney disease screening was suboptimal, medication access was limited, and adherence to national guidelines was low. Short consultation times, and staff shortages further hindered care delivery. Three major barriers were identified, highlighting key areas for targeted improvement.

Studies on barriers of DM and hypertension management in Indonesia are limited and mostly focused on patient perspectives, demonstrating issues including low health literacy, poor adherence, financial and transportation obstacles, and low PROLANIS engagement (16–19). However, provider- and system-level barriers—despite their significant impact on care quality—remain underexplored.

A major challenge in managing diabetes and hypertension is poor understanding of referral practices–affecting 84.5% providers. Delays in clinically indicated referrals (16) are mainly due to insufficient training, leading to low awareness of early referral benefit (20). A study from the US reported that the unclear roles and responsibilities of the primary health care providers were also complicated the referral management (21). System-level barriers also play role, including inefficient and fragmented health information systems. Delays are common with platforms like the Integrated Referral Information System (SISRUTE) and the Integrated Emergency Management System (SPGDT), due to slow response times, poor internet access, and complex user interfaces (20). Sperati et al. also reported that poor compliance with current guidelines contributes to the problem (22). Additionally, unclear administrative procedures and patients' concern about costs and transportation further limit access (22–25). These challenges were consistently confirmed in interviews with healthcare providers (Table 3).

Misconceptions were prevalent: 77.6% of providers believed referrals were unnecessary if resources were sufficient, and 44.8% selected unrelated referral conditions, demonstrating misalignment with clinical guidelines (13, 14, 26, 27). Accurate referral criteria are essential to ensure timely, appropriate, and cost-effective care (27). Similar observation reported from a study in the Middle East, which found that although primary care providers retain good awareness and knowledge, the gap between knowledge and how referrals were actually implemented in practice remains a concern (28).

While 75.4% of subjects correctly identified hypertension referral criteria, 24.6% preferred to refer patients with sudden blood pressure rise in uncomplicated essential hypertension. This reflects a disconnect between clinical guidelines and practice, partly due to limited training and restricted drug availability in primary care. Most centers had access only to calcium-channel blockers and RAAS-inhibitors, with no intravenous options. While referrals may be appropriate in hypertensive emergencies, they are unnecessary in uncomplicated cases. The widespread use of calcium-channel blockers likely stems from their first-line status in guidelines, affordability, and prescriber familiarity. However, consistent access to RAAS-inhibitors is also important, given their proven cardiovascular benefits. Enhancing primary care providers' familiarity with relevant clinical guidelines and strengthening their ability to appropriately use RAAS inhibitors in patient management are key areas for improvement (29, 30).

Our study identified significant knowledge gaps on PROLANIS, particularly around its target population and outcome goals. These misunderstandings risk inappropriate patient enrolment and reduce program effectiveness. Inconsistent awareness of performance targets also reflects broader communication issues. Several factors contribute to suboptimal PROLANIS implementation (see Table 3), including personnel shortage, insufficient equipment and laboratory infrastructure, and financial challenges (31, 32). Delayed BPJS reimbursements and bureaucratic deferrals have disrupted program continuity (33). Systemic issues such as poor coordination, unclear performance indicators, and low adherence to national guidelines further undermine the program (32). The COVID-19 pandemic exacerbated these problems by diverting resources and limiting face-to-face services (34).

Significant knowledge gap in diagnosis and management of diabetes was also identified. This aligns with national survey report, showing declining diabetes knowledge, particularly among graduates from newer, less-regulated institutions, raising concerns about preservice education quality (35). Limited in-service training, inadequate infrastructure, resource shortages, heavy workloads, and low provider engagement further compromise effective diabetes care at primary level (36–40).

This study identified key barriers to chronic disease management in primary care. Patient barriers included low health literacy, limited engagement, and logistical issues like long travel distances, which delay diagnosis, treatment, and adherence—worsening outcomes. Provider barriers involved gaps in understanding clinical guidelines, referral protocols, and PROLANIS program, highlighting the need for targeted training and continuing professional development. Structural challenges included inefficient administration, long wait times, lack of functional referral systems, poor clinical information integration, and limited referral coordination staff. Addressing these barriers is crucial for more effective, patient-centered chronic disease care.

To our knowledge, this is the first study to provide an in-depth evaluation of perceived barriers to chronic disease management in Indonesia. Using a mixed-methods approach, the study identified key themes—including systemic constraints, resource limitations, patient-related challenges, and provider attitudes—that shape the delivery of care in primary care settings. Importantly, the comparison between healthcare providers' perceptions and those previously reported by service users offers valuable insight: while users often highlight issues such as long waiting times, limited continuity of care, and difficulties navigating the referral system, providers tend to emphasize structural barriers, administrative burdens, and gaps in patient adherence. This divergence in perspectives underscores the need for interventions that address both sides of the care experience, ensuring that policy and programmatic solutions are informed not only by health system requirements but also by patient expectations and lived experiences. These findings therefore provide critical context for designing targeted interventions and strengthening policies to improve chronic disease management in Indonesia.

### Study limitations

However, this study has limitations. The questionnaire was not pilot-tested across the full range of healthcare professional categories included in the study. Although the items were developed using general medical language and Bahasa Indonesia to ensure broad comprehensibility, we cannot fully exclude the possibility that differences in educational background may have influenced interpretation of certain questions. Results may not be fully generalizable to all healthcare providers or regions as a relatively small and non-random sample, and conclusions are based on self-reported data rather than direct observation. Nonetheless, consistency across study sites strengthens the credibility of the findings.

## Conclusion

This study highlights significant knowledge gaps among primary healthcare providers in Indonesia in managing chronic conditions like diabetes and hypertension. Combined with patient-related barriers, limited provider training, and systemic inefficiencies, these issues complicate effective care delivery. Improving outcomes requires targeted education, strengthen referral systems, and better implementation of national programs such as PROLANIS.

## Data Availability

The original contributions presented in the study are included in the article/Supplementary Material, further inquiries can be directed to the corresponding author.
