# Supplementary material for: Perceived barriers to management of patients with diabetes mellitus and hypertension in primary care centers in Indonesia
Source: Front Health Serv. 2026 Jan 15;5:1715125. doi: 10.3389/frhs.2025.1715125 (PMC12852415; doi:10.3389/frhs.2025.1715125)
Supplement: Supplementary file 1 [file Table1.docx]

SUPPLEMENTARY MATERIALS

**Table S1. List of questions on the referral knowledge**

| RK1 | Do you know that every first-level health facility in running its services is obliged to implement a referral system in accordance with the laws in force? | **Yes** |
| --- | --- | --- |
|  |  | No |
| RK2 | Based on PERKENI 2021, T2DM patients at primary healthcare need to be referred to secondary health facilities if...  *PERKENI: the Indonesian society of diabetes and endocrinology | GDP>130 mg/dL and GDPP>180 mg/dL found in the last 6 months |
|  |  | **Not achieving blood sugar targets when on OAD monotherapy for 3 months** |
|  |  | Chronic DM complications during the last 6 months |
|  |  | HbA1C = 6.5 |
| RK3 | Criteria for emergency in DM patients who must be referred to secondary/tertiary health care based on PERKENI  *PERKENI: the Indonesian society of diabetes and endocrinology | Patients with a change in medication history during consecutive weeks |
|  |  | Patients with drastic weight gain |
|  |  | **Patients with ketoacidosis** |
| RK4 | The law permits both horizontal and vertical referrals for primary health care. | **Yes** |
|  |  | No |
| RK5 | Vertical referral is not possible when... | **The patient's condition needs to be treated without the need for specialized measures** |
|  |  | Facilities, equipment and healthcare workers available at primary health care facilities |
|  |  | The second/third health care level are provided with better resources compare to the first level |
| RK6 | Which illnesses are eligible for referral to advanced healthcare facilities? | Hepatitis A |
|  |  | Dyslipidaemia |
|  |  | Preeclampsia |
|  |  | DM type 1 and 2 |
|  |  | **DM complications** |
| RK7 | What is the indication to refer a hypertensive patient to the advanced healthcare facilities | Sudden onset of hypertension with a history of normal blood pressure |
|  |  | Essential hypertension patients |
|  |  | The patient's clinical condition not yet requiring specialistic management |
|  |  | **Patients with hypertension mediated organ damage** |
| RK8 | PROLANIS is a program at primary care to improve the quality of life for patients with chronic illnesses  *PROLANIS: program pengelolaan penyakit kronis (Management of chronic disease program), established by the national health insurance (BPJS) | **Yes** |
|  |  | No |
| RK9 | Who is the target of the PROLANIS program? | BPJS participants with controlled hypertension and obesity |
|  |  | BPJS participants with controlled hypertension and DM T1 |
|  |  | **BPJS participants with controlled hypertension and DM T2** |
| RK10 | Minimum target of RPTT (the ratio of Controlled PROLANIS Participant) is... | **5%** |
|  |  | 10% |
|  |  | 15% |
|  |  | 20% |

*Text highlight in bold presenting the correct answer.

**Table S2. List of questions on the hypertension knowledge**

| HK 1 | Definition of hypertension | **Correct** |
| --- | --- | --- |
|  |  | False |
| HK 2 | Hypertension is always associated with specific symptoms | Correct |
|  |  | **False** |
| HK 3 | Patients are more likely to have hypertension if their parents also have hypertension | **Correct** |
|  |  | False |
| HK 4 | High systolic blood pressure is more likely to cause heart disease than diastolic blood pressure | Correct |
|  |  | **False** |
| HK 5 | Blood Pressure measurements are taken at least 2 times per examination with an interval of 5 minutes | **Correct** |
|  |  | False |
| HK 6 | Blood pressure measurements should be re-examined if there is a difference in mean results of more than 5 mmHg | **Correct** |
|  |  | False |
| HK 7 | Confirmation of the diagnosis of hypertension cannot only rely on 1 examination, unless the patient's blood pressure is very high | **Correct** |
|  |  | False |
| HK 8 | Overweight patients are 2-6 times more likely to have hypertension | **Correct** |
|  |  | False |
| HK 9 | Regular physical activity helps lower blood pressure | **Correct** |
|  |  | False |
| HK 10 | A young person will not have hypertension | Correct |
|  |  | **False** |
| HK 11 | Hypertension is harmful to the body because it will... | Leads to weight gain |
|  |  | **Causes damage to blood vessels** |
|  |  | Causes nervousness/anxiety |
| HK 12 | Why is hypertension known as the silent killer? | Low risk of death due to hypertension |
|  |  | When having pain and not having pain it still feels fine |
|  |  | **When suffering from high blood pressure it may be asymptomatic but can be health-threatening** |
| HK 13 | Patients are diagnosed with hypertension if... | Continuous headache for 6 months |
|  |  | Having family members with hypertension |
|  |  | Stress |
|  |  | **Blood pressure above normal limits on measurements at 3 different times** |
| HK 14 | Which is the most appropriate treatment for hypertension? | **Additional hypertension medications may be given when blood pressure increases further** |
|  |  | Medication should not be taken with alcohol |
|  |  | Daily consumption of hypertension medications will damage other organ functions |
| HK 15 | All of the following health problems can be caused by high blood pressure, except... | Heart attack |
|  |  | Stroke |
|  |  | **Gout** |
|  |  | Kidney disease |
| HK 16 | According to the consensus on hypertension management, the normal blood pressure target for hypertensive patients aged >65 is.... | **130-139/70-79 mmHg** |
|  |  | <120/<80 mmHg |
|  |  | 140/90 mmHg |
|  |  | >140/>90 mmHg |
| HK 17 | A sign that a patient is experiencing symptoms of kidney failure is... | Heartburn |
|  |  | **Water retention** |
|  |  | Polyuria |
|  |  | Hair fall |
| HK 18 | An examination that is not required to identify kidney failure is... | Creatinin |
|  |  | eGFR |
|  |  | Albuminuria |
|  |  | **APTT** |
| HK 19 | When do you decide to refer a patient with signs of kidney failure? | If there is a decrease in albumin levels |
|  |  | If the patient's eGFR is 60-89 ml/min/1.73 m² |
|  |  | **If the patient's eGFR is <30 ml/min/1.73 m²** |
|  |  | If the patient's eGFR is 90 ml/min/1.73 m² |
| HK 20 | Elevated potassium levels can be dangerous because... | Leads to an increase in excess body fluids |
|  |  | Leads to an increase in hematocrit levels |
|  |  | Leads to an increased production of white blood cells |
|  |  | **Causes an irregular heart rhythm** |
| HK 21 | Drugs that should be avoided for patients with history of chronic kidney disease are... | Lisinopril |
|  |  | Tylenol |
|  |  | **NSAID** |
|  |  | Sodium bicarbonate |

*Text highlight in bold presenting the correct answer.

**Table S3. List of questions on the diabetes knowledge**

| DK 1 | Based on the causes, DM is categorized into 2 categories | Correct |
| --- | --- | --- |
|  |  | **False** |
|  |  | Not sure |
| DK 2 | The best time to check blood sugar is 2 hours before and after eating | **Correct** |
|  |  | False |
|  |  | Not sure |
| DK 3 | Symptoms of hypoglycemia in diabetic patients are trembling, sweating and confusion | **Correct** |
|  |  | False |
|  |  | Not sure |
| DK 4 | Patients with type 1 DM who consume food should have blood sugar monitoring every 6 hours | Correct |
|  |  | **False** |
|  |  | Not sure |
| DK 5 | Self-monitoring of blood glucose is recommended for patients on daily injection insulin treatment | **Correct** |
|  |  | False |
|  |  | Not sure |
| DK 6 | The side effects of insulin therapy are hyperglycemia | Correct |
|  |  | **False** |
|  |  | Not sure |
| DK 7 | DM treatment aimed at lowering HbA1C only | Correct |
|  |  | **False** |
|  |  | Not sure |
| DK 8 | If the patient's parents have diabetes, the patient is more likely to have diabetes as well | **Correct** |
|  |  | False |
|  |  | Not sure |
| DK 9 | Physical exercise is one of the pillars in the management of type 2 DM without nephropathy | **Correct** |
|  |  | False |
|  |  | Not sure |
| DK 10 | The diagnosis of DM can be confirmed by the presence of glucosuria | Correct |
|  |  | **False** |
|  |  | Not sure |
| DK 11 | The overweight group (BMI >23 kg/m²) is at greater risk of developing diabetes if one of the following factors is present | HDL < 35 and/or triglycerides >200 |
|  |  | **History of cardiovascular disease** |
|  |  | No history of pre-diabetes |
| DK 12 | Hypoglycemia can be seen in various conditions | **Glucose at random < 70 mg/dL** |
|  |  | Glucose at random > 70 mg/dL |
|  |  | Alcohol consumption with the right meal |
| DK 13 | All of the following health problems can be caused by DM, except... | Retinopathy |
|  |  | Kidney disease |
|  |  | **Arthritis** |
| DK 14 | Early detection of foot abnormalities in diabetic patients can be done if the following examination results are found | **There is a clavus/fish eye on the sole of the foot** |
|  |  | Rough skin but not scaly and cracked |
|  |  | The toe bones do not protrude |
| DK 15 | In TTGO (Oral Glucose Tolerance Test) screening with 75 g of glucose, the patient is immediately said to have gestational diabetes if the blood glucose value is... | Preprandial glucose > 90 mg/dL |
|  |  | Glucose after 1 hour > 92 mg/dL |
|  |  | HbA1C > 7% |
|  |  | **Glucose after 2 hour > 153 mg/dL** |
| DK 16 | Pregnant female type 2 DM patients are recommended to have a blood glucose target of... | Preprandial glucose : 60-80 mg/dL |
|  |  | **Preprandial glucose : 100-120 mg/dL** |
|  |  | Preprandial glucose before and after meal: 80-120 mg/dL |
|  |  | Glucose after 1 hour after meal: 100-155 mg/dL |

*Text highlight in bold presenting the correct answer.

**Table S4. List of questions on the health service provided in primary care center**

| Are all of medicines covered by BPJS? | Yes |
| --- | --- |
|  | No |
| In treating patients, how often do you follow the technical guidelines for the management of patients with DM/HT? (PB PERKENI and Ministry of Health guidelines) | Always |
|  | Frequently |
|  | Rarely |
|  | Never |
| How often do you educate patients about the side effects of medications and how to minimize them? | Always |
|  | Frequently |
|  | Rarely |
|  | Sometimes/never |
| Do you frequently ask your patients about medication adherence? | Always/every meeting |
|  | Frequently/few times |
|  | Rarely/occasionally |
|  | Sometimes/never |
| Besides medication, have you ever educated patients on HT/DM diet management? | Yes |
|  | No |
| How often do you educate patients on how to check their blood sugar at home? | Always/every meeting |
|  | Frequently/few times |
|  | Rarely/occasionally |
|  | Sometimes/never |
| How often do you educate patients to do blood pressure checks at home? | Always/every meeting |
|  | Frequently/few times |
|  | Rarely/occasionally |
|  | Sometimes/never |
| What blood sugar targets do you give to patients with diabetes? | Preprandial glucose 80-130 mg/dL |
|  | Preprandial glucose <200 mg/dL |
| What target blood pressure do you give to patients with hypertension? | BP <150/90 mmHg |
|  | BP <140/90 mmHg |
| What percentage of HT and DM patients in this public health care center are controlled/achieved treatment targets? | 81%-100% |
|  | 51%-80% |
|  | 31%-50% |
|  | 0%-30% |
| Do you have any other job besides working at the public health center? | Yes |
|  | No |
| How long is the duration of face-to-face meetings per patient? | > 10 minutes per patient |
|  | < 10 minutes per patient |
| In your opinion, is the incentive/salary given in line with your current workload? | Yes |
|  | No |
| What challenges do puskesmas most often experience in handling patients? | Limited human resources |
|  | BPJS system |
|  | Facilities and infrastructure that are less supportive |
|  | Limited service hours |
|  | Others |
| How often do you perform urinalysis and renal Ur/Cr tests on patients with HT and DM? | Very often |
|  | Often |
|  | Rarely |
|  | Never |


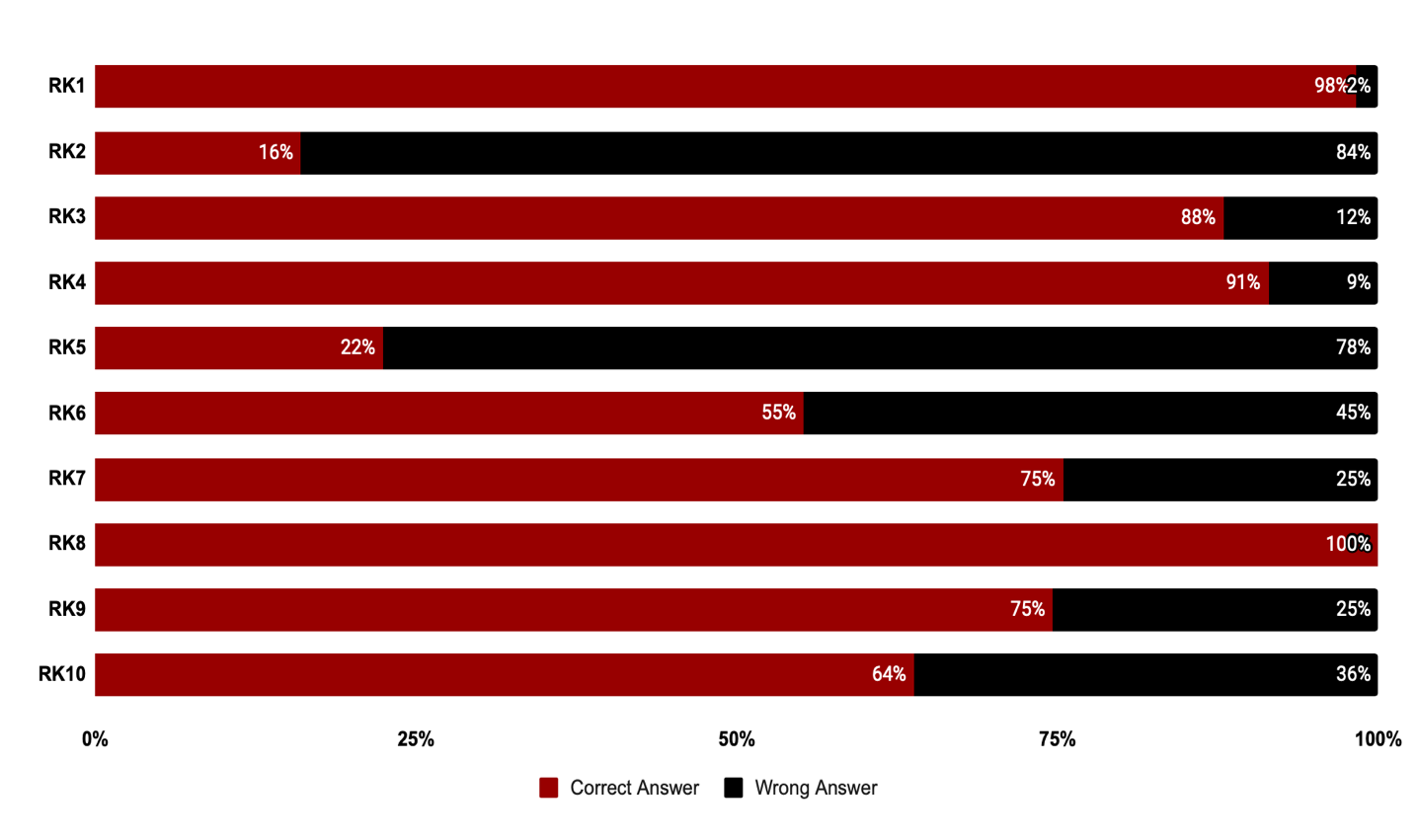


**Supplementary Fig 1. Distribution on the referral knowledge (RK) of the subjects**


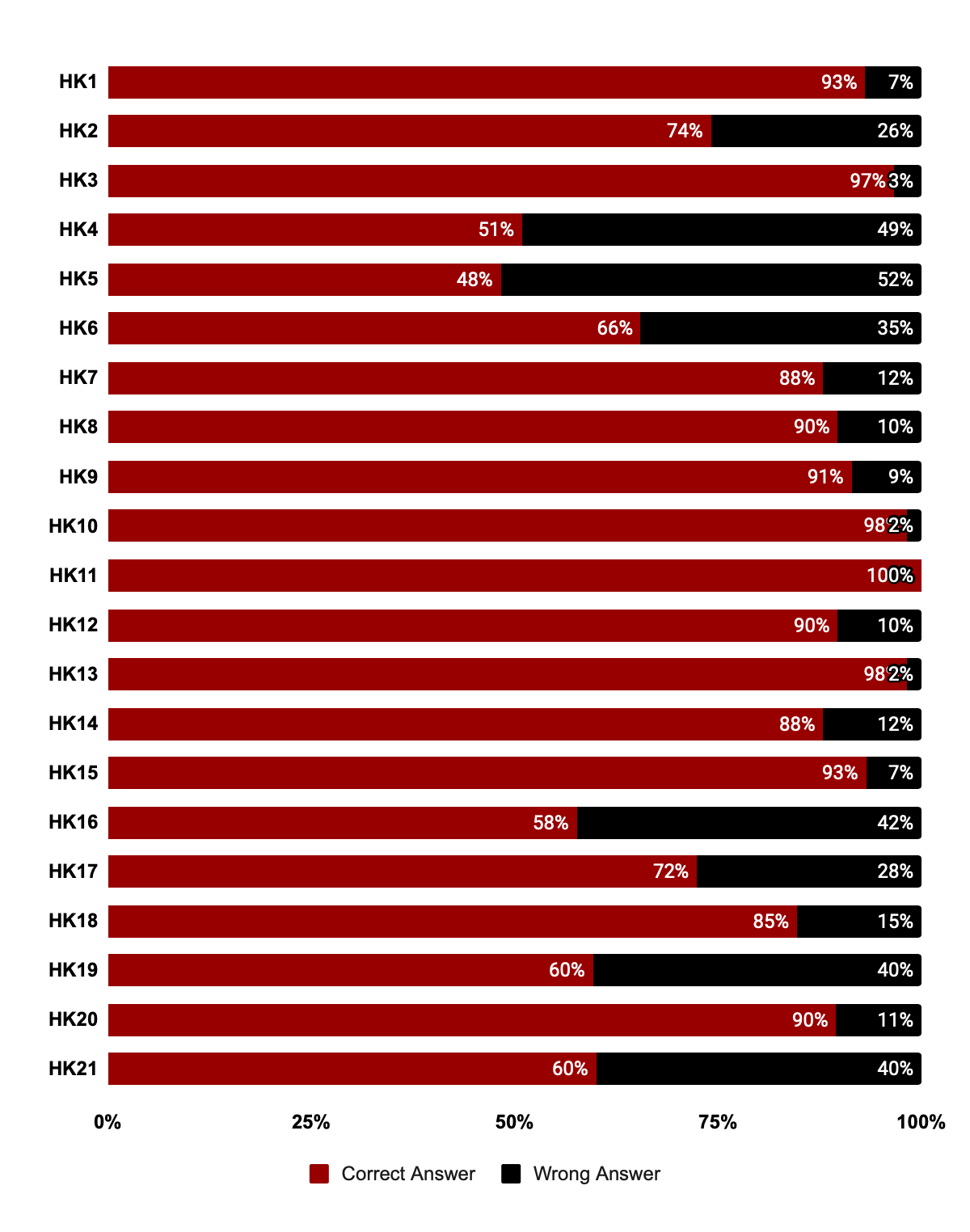


**Supplementary Fig 2. Distribution on the hypertension knowledge (HK) of the subjects**


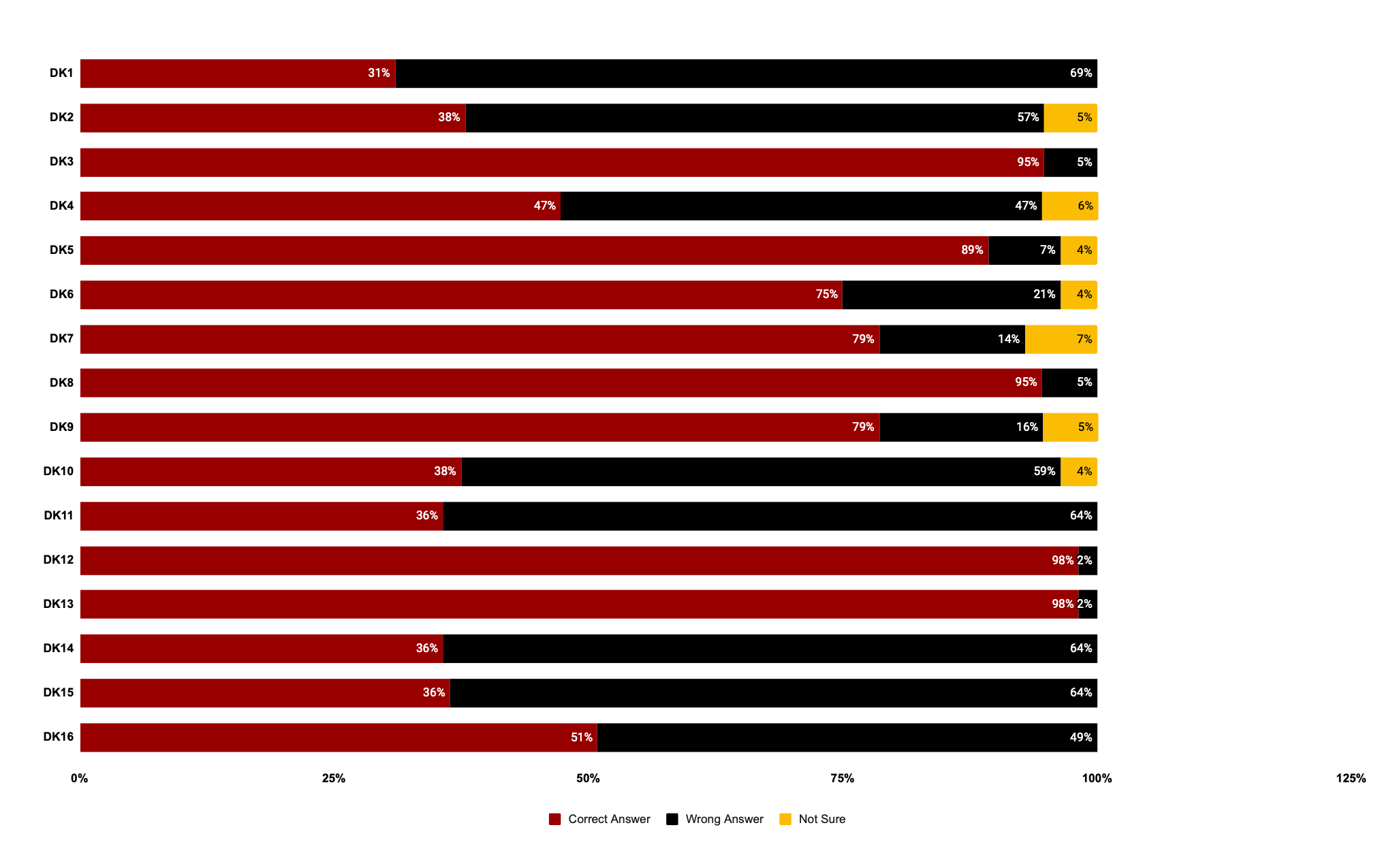


**Supplementary Fig 3. Distribution on the diabetes knowledge of the subjects**


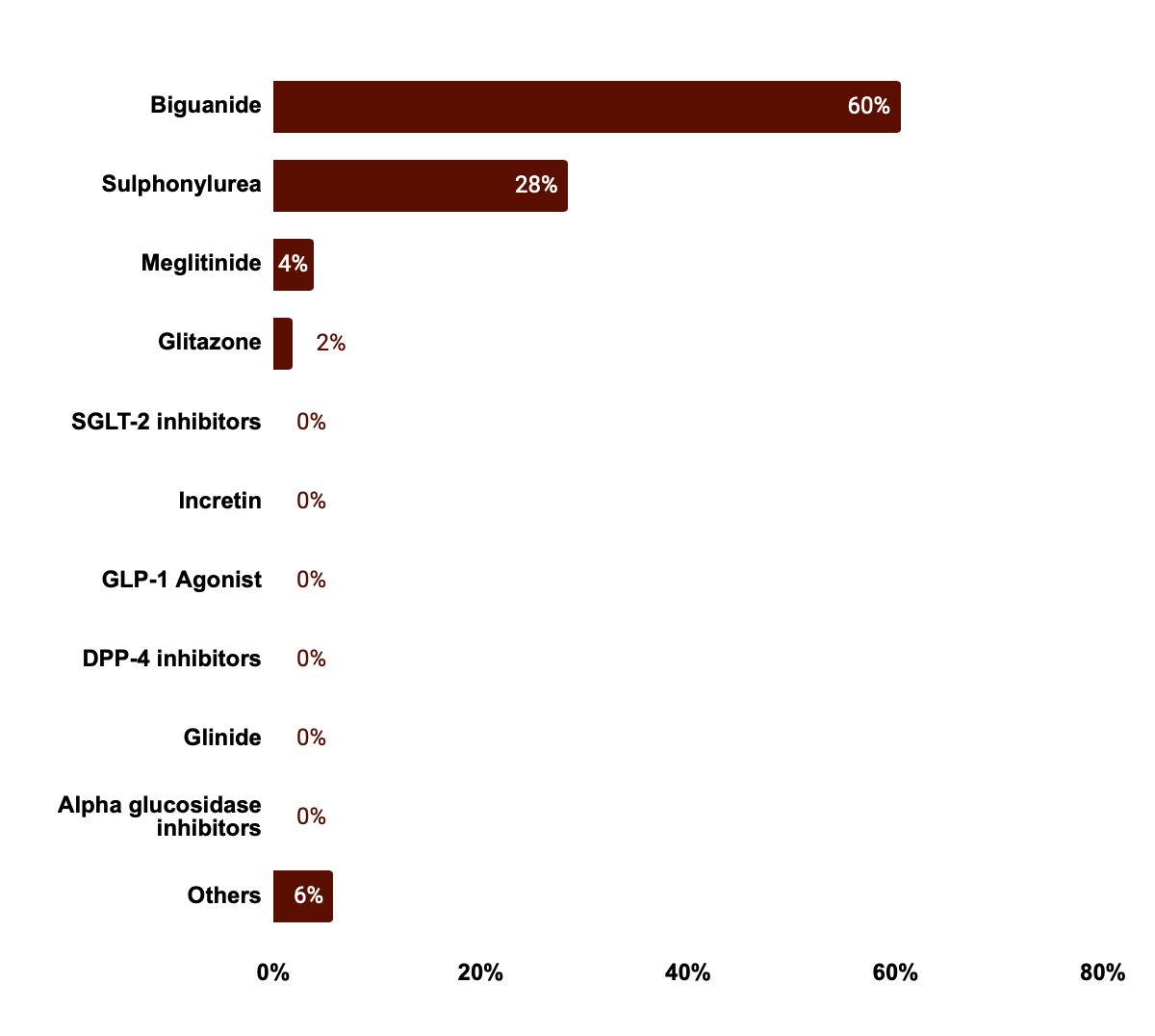


**Supplementary Fig 4. Oral anti-diabetes drugs available in primary care centers**


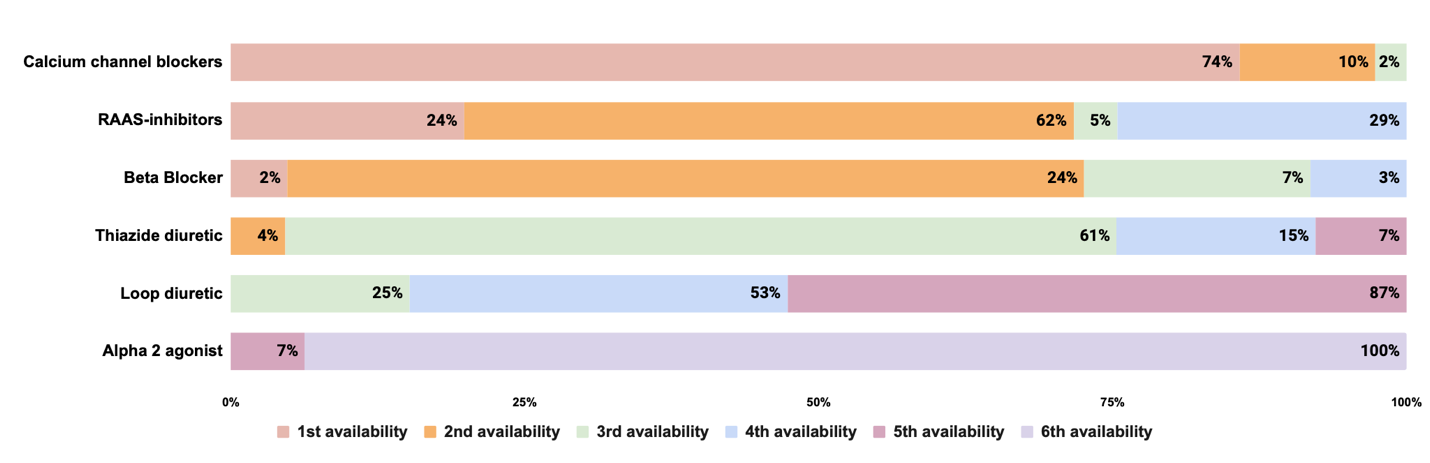


**Supplementary Fig 5. Availability of oral antihypertensive agents in primary care facilities**
